# Repurposing of Drug Candidates for Treatment of Skin Cancer

**DOI:** 10.3389/fonc.2020.605714

**Published:** 2021-01-08

**Authors:** Hernán Cortés, Octavio D. Reyes-Hernández, Sergio Alcalá-Alcalá, Sergio A. Bernal-Chávez, Isaac H. Caballero-Florán, Maykel González-Torres, Javad Sharifi-Rad, Manuel González-Del Carmen, Gabriela Figueroa-González, Gerardo Leyva-Gómez

**Affiliations:** ^1^ Laboratorio de Medicina Genómica, Departamento de Genómica, Instituto Nacional de Rehabilitación Luis Guillermo Ibarra Ibarra, Ciudad de México, Mexico; ^2^ Laboratorio de Biología Molecular del Cáncer, UMIEZ, Facultad de Estudios Superiores Zaragoza, Universidad Nacional Autónoma de México, Ciudad de México, Mexico; ^3^ Facultad de Farmacia, Universidad Autónoma del Estado de Morelos, Cuernavaca, Morelos, Mexico; ^4^ Departamento de Farmacia, Facultad de Química, Universidad Nacional Autónoma de México, Ciudad de México, Mexico; ^5^ CONACyT-Laboratorio de Biotecnología, Instituto Nacional de Rehabilitación Luis Guillermo Ibarra Ibarra, Ciudad de México, Mexico; ^6^ Phytochemistry Research Center, Shahid Beheshti University of Medical Sciences, Tehran, Iran; ^7^ Facultad de Medicina, Universidad del Azuay, Cuenca, Ecuador; ^8^ Facultad de Medicina, Universidad Veracruzana, Ciudad Mendoza, Veracruz, Mexico; ^9^ Laboratorio de Farmacogenética, UMIEZ, Facultad de Estudios Superiores Zaragoza, Universidad Nacional Autónoma de México, Ciudad de México, Mexico

**Keywords:** skin cancer, drug repurposing, melanoma, nanocarriers, drug delivery systems

## Abstract

Skin cancers are highly prevalent malignancies that affect millions of people worldwide. These include melanomas and nonmelanoma skin cancers. Melanomas are among the most dangerous cancers, while nonmelanoma skin cancers generally exhibit a more benign clinical pattern; however, they may sometimes be aggressive and metastatic. Melanomas typically appear in body regions exposed to the sun, although they may also appear in areas that do not usually get sun exposure. Thus, their development is multifactorial, comprising endogenous and exogenous risk factors. The management of skin cancer depends on the type; it is usually based on surgery, chemotherapy, immunotherapy, and targeted therapy. In this respect, oncological treatments have demonstrated some progress in the last years; however, current therapies still present various disadvantages such as little cell specificity, recurrent relapses, high toxicity, and increased costs. Furthermore, the pursuit of novel medications is expensive, and the authorization for their clinical utilization may take 10–15 years. Thus, repositioning of drugs previously approved and utilized for other diseases has emerged as an excellent alternative. In this mini-review, we aimed to provide an updated overview of drugs’ repurposing to treat skin cancer and discuss future perspectives.

## Introduction

Skin cancers are highly prevalent malignancies worldwide, ranked at the twentieth place of incidence (1, 2). There were an estimated 100,000 new melanoma cases in the United States during 2020, with the approximate death of 6,850 people. The prevalence is higher in men, and the incidence varies according to the geographic region and by country (3). Skin cancers include melanomas and nonmelanoma skin cancers (NMSC). Melanomas are tumors that develop in melanocytes, and these may appear in diverse body regions. Specialists consider melanoma one of the most dangerous cancers (4). Patients in advanced stages commonly have a discouraging prognosis, and the five-year survival in those patients is <5%. Remarkably, patients without treatment exhibit a median survival between six and nine months (5). The main types of NMSC include basal cell carcinoma (BCC) and squamous cell carcinoma (SCC). NMSCs have a higher occurrence than melanoma, but they are less lethal, especially if diagnosed early (6). BBCs are skin tumors produced by the abnormal growth of basal cells. It is the most frequent type of skin cancer (7), and they are curable in many cases when detected timely. On the other hand, SCC is the second most frequent skin cancer type; it develops in the squamous cells located in the epidermis (8). SCC generally exhibits a benign clinical pattern; notwithstanding, it may sometimes be aggressive and metastatic (9).

Skin cancers develop more frequently in body regions exposed to the sun; however, they may also appear in areas that do not usually get sun exposure. Thus, their development is multifactorial, comprising endogenous (skin type and genetic factors) and exogenous (degree of sun exposure and sun protection conduct) risk factors (10). Among exogenous aspects, ultraviolet radiation (UVR) is the most notable risk factor. UVR can produce DNA damage, mutations, inflammatory responses, and oxidative stress, leading to skin cancer development (11). Among the UVR types, ultraviolet A (UVA) penetrates deeper into the skin, producing more considerable skin damage than the ultraviolet B (UVB). Nevertheless, UVB is mostly related to inflammatory responses and DNA damage as a critical tumor-promoting event (12).

Skin cancer management depends on the type; it is usually based on surgery, chemotherapy, immunotherapy, and targeted therapy (7, 9, 13, 14). In this respect, oncological treatments have demonstrated some progress in the last years; however, current therapies still present various disadvantages such as little cell specificity, recurrent relapses, high toxicity, and increased costs (14). Furthermore, the pursuit of novel medications is expensive, and the authorization for their clinical utilization may take 10–15 years (15). Thus, repositioning drugs previously approved and utilized for other diseases has emerged as an excellent alternative (16). In this mini-review, we aimed to provide an updated overview of drugs’ repurposing to treat skin cancer and discuss future perspectives.

## Drug Repurposing for Skin Cancer

Drug repurposing is the process of giving new applications for existing drugs; it may considerably diminish development costs (and times) to search for effective strategies to treat skin cancer (17). Repurposing drugs possess various advantages, including data availability about clinical tests, chemical composition, and possible toxicity, which can accelerate their application in clinical trials (18). Although various drugs have been proposed for their repurposing in skin cancer (**Table 1**), most of them have only been evaluated in preclinical studies, and extensive clinical trials are needed before their approval for skin cancer treatment. Nonetheless, these drugs represent a promising alternative because almost all are cheap and without significant adverse effects on therapeutic doses. Drugs that have been suggested for repositioning in skin cancer are discussed in the next sections in alphabetical order as an example of the most prominent proposals to date.

**Table 1:** Drugs proposed for chemoprevention and treatment of skin cancer.

Drug	Other uses	Study model	Mechanism of action	Reference
Albendazole	Useful for giardiasis, trichuriasis, filariasis, neurocysticercosis, among other diseases	A375 and A2058 metastatic melanoma cells lines	Induction of DNA damage and cells arrest in the G2/M phase of the cell cycle, sensitizes them to radiation therapy	(19)
Desmopressin	Synthetic hormone commonly used for nocturia and enuresis	Mice xenografted with B16-F0 melanoma cells	Modulation of proteolysis and coagulation	(20)
Digoxin	Antiarrhythmic agent used in heart failure, and other heart disorders such as atrial fibrillation	Primary melanocytes (hMEL1) or melanoma cells derived from patients	Inhibits the ATP1A1 Na þ/K þ pump, which is highly expressed by melanoma	(21)
Doxycycline	Antibiotic used to treat infections such as skin infections and rosacea. It can also be used to prevent malaria	Human (A2058 and A375) and mouse (B16F10) melanoma cells	Inhibition of the MMP-2 and MMP-9 metalloproteinases activity, activation of apoptosis signal-regulated kinase 1, c-Jun N-terminal kinase, and caspases, which induces apoptosis	(22, 23)
Fenofibrate	Used with other medications to reduce fatty substances such as cholesterol and triglycerides	Human (SkMell88) and mouse (B16F10) melanoma cells	Anti-metastatic activity involving down-regulation of Akt phosphorylation	(24)
Flubendazole	Anthelmintic drug used in parasitic infestations	A375, BOWES, and RPMI-7951 cells	Anti-melanoma activity related to enhanced transcription of p53 and NF-ĸB, as well as phosphorylation of JNK	(25)
Haloprogin	Antifungal drug used to treat skin infections such as athlete’s foot	Mouse B16F10 skin melanoma tumor model	In combination with RAPTA-T, shown to be a profitable candidate for its use as a melanoma growth inhibitor through cancer cell death induction	(26)
Itraconazole	Used to treat fungal infections as aspergillosis, blastomycosis, and coccidioidomycosis	SK-MEL-28 and A375 human melanoma cells	Inhibits the proliferation and colony formation through the Hh, Wnt, and PI3K/mTOR signaling pathways blockade	(27)
Leflunomide	Used in active moderate-to-severe rheumatoid arthritis and psoriatic arthritis	Human melanoma cell lines	A selective inhibitor of *de novo* pyrimidine synthesis, blocking the synthesis of DNA and RNA; reduces cell proliferation and causes cells to arrest in G1 of the cell cycle.	(28)
Lidocaine	Local anesthetic and antiarrhythmic	Human keratinocytes	It induces membrane permeability and excessive production of reactive oxygen species (ROS).	(29)
Mebendazole	Used to treat parasitic worm infestations as ascariasis, worm infections, and giardia, among others	Human melanoma cell lines	Bcl-2 phosphorylation in melanoma cells, avoiding its interaction with pro-apoptotic Bax, through apoptosis induction	(30)
Metformin	Commonly used to treatment of type 2 diabetes, also used in the treatment of polycystic ovary syndrome	Human melanoma cell lines	Induces cell cycle arrest in the G0-G1 phase, and it´s responsible for autophagy and apoptosis induction	(31, 32)
Naproxen	Used to treat pain, menstrual cramps, inflammatory diseases such as rheumatoid arthritis, and fever	Mice irradiated with UVB	Reduction in the incidence of tumor lesions by naproxen may be due to its ability to increase TNF-a levels and decrease PGE2.	(33)
Niclosamide	Anti-helminthic drug, has been used to treat tapeworm infection	In vitro: human and mouse melanoma cell lines. In vivo: a mouse xenograft model of A375 cell line	Induces cell apoptosis *via* the mitochondrial-mediated apoptotic pathway, also inhibits tumor growth by decreasing the expression of p-STAT3, MMP-2, and MMP-9	(34)
Nicotinamide	Treatment and prevention of niacin deficiency and certain conditions related such as pellagra	Human keratinocytes	Chemopreventive effects, replenishes cellular ATP after UV irradiation, and enhancement of DNA repair in UV-irradiated human skin	(35)
Pimozide	Decreasing the activity of a natural substance (dopamine) in the brain, Tourette syndrome patients	B16 cell-bearing mice	Antitumor activity *via* the regulation of proliferation, apoptosis, and migration	(36)
Piroxicam	Used to the treatment of rheumatoid arthritis and osteoarthritis, acute musculoskeletal disorders, and dysmenorrhea	Patients affected by Actinic keratoses	A non-selective NSAID* that inhibits the activity of COX-1 and COX-2, inducing apoptosis and inhibit recruitment and production of growth factors and other carcinogenetic mediators	(37, 38)
Propranolol	β-blocker commonly used for high blood pressure	Patients with melanoma	Inhibition of angiogenesis and migration of cancer cells	(39)
Rafoxanide	Anthelmictic used mainly for the treatment of *fasciola hepatica*	A375 and A431 cells and mice xenografted with those cells	Inhibition of CDK4/6, increase of cell apoptosis, and arrest of cell cycle	(40)
Telmisartan	Angiotensin receptor 1 inhibitor widely used as an antihypertensive drug	Human melanoma cells A375, 518a2, and HTB140	Induction of apoptosis, generation of reactive oxygen species, and alteration of cell bioenergetics	(41)

*NSAID, Non-Steroidal Anti-Inflammatory Drugs.

### Digoxin

Digoxin is a compound utilized to treat arrhythmia and heart failure symptoms. Its mechanism of action includes inhibition of the hypoxia-inducible factor-1α (HIF-1α) (42), which contributes to angiogenesis, metastasis, and tumor resistance in many cancers (43). Concerning this, Eskiocak et al. (21) suggested a therapeutic effect of digoxin against melanoma. The authors reported that digoxin exhibited low cytotoxic activity in mice xenografted with metastatic melanomas derived from patients. However, the authors found a synergistic beneficial effect when simultaneously administered with a MEK inhibitor, extending experimental mice’s survival. The possible mechanism of action included acidification of cytoplasm, rises in mitochondrial Ca^2+^ levels, depletion of ATP, and mitochondrial function reduction. Likewise, the combination of digoxin and DMXAA (an anti-vascular agent) inhibited tumors’ regrowth in mice harboring B16F10 melanoma tumors (44). The enhancement in the efficacy may be explained by the inhibition of HIF-1α and stimulation of the immune function. Concerning human studies, a recent clinical trial explored the effects of parallel administration of digoxin and trametinib on BRAF wild-type metastatic melanoma patients (45). The results exposed a reasonable rate of control of disease in those patients for up ten months. Thus, this approach could be useful in metastatic melanoma patients refractory or intolerant of immunotherapy; nonetheless, additional clinical trials with a higher number of patients will be crucial.

### Doxycycline

Doxycycline is a broad-spectrum tetracycline antibiotic (46). Some studies reported that doxycycline might inhibit several matrix metalloproteinases that participate in diverse cancers’ metastasis (47). Thus, it has been suggested that this drug could be repositioned as an anti-cancer treatment (48). An interesting study demonstrated that doxycycline inhibited the growth of melanoma cells (49). The anti-tumor effects might be mediated by various mechanisms, including inhibition of the NF-ĸB pathway, decrease of antiapoptotic proteins, cytochrome C release, and activation of caspase-8 (50). Likewise, doxycycline inhibited the adhesion and migration of a melanoma cell line, with subsequent apoptosis induction (51). This activity appeared to be mediated by inhibition of focal adhesion kinase, which participates in migration and cell adhesion regulation. Likewise, a very recent study showed that doxycycline diminished the viability and proliferation of a melanoma cell line (COLO829 cells) by decreasing intracellular levels of reduced thiols and impairing the homeostasis of the cells (22). Finally, a recently finished clinical trial found that the concomitant administration of doxycycline, temozolomide, and ipilimumab produced no significant clinical improvement in patients with melanoma (NCT01590082). Although this finding could appear disappointing, preclinical studies suggest a therapeutic usefulness of doxycycline and warrant further clinical trials.

### Fenofibrate

Fenofibrate is an agonist of the peroxisome proliferator-activated receptor-α; it is indicated for managing mixed dyslipidemia and hypertriglyceridemia (52). A variety of studies have reported that fenofibrate exerts anti-tumor activities in several cancers (53, 54), including melanoma (55, 56). Panigraphy et al. (56) exposed that fenofibrate significantly inhibited the proliferation of melanoma cells (B16-F10 cells) and suppressed primary tumors’ growth *in vivo* in a murine model. Those effects were mediated by the inhibition of inflammation and angiogenesis in the surrounding host tissue. Additionally, fenofibrate significantly decreased melanoma metastases when administered *via* oral in mice, suggesting that this compound possesses chemopreventive activity (55). Interestingly, a down-regulation in the phosphorylation of Akt might explain this anti-metastatic effect (24). Finally, a very recent study proposed that the effects of fenofibrate on growth and metastases of melanoma could be produced by inhibiting the TLR4-dependent signaling pathway (57). Despite the studies suggesting beneficial effects of fenofibrate in melanoma, currently, there are no ongoing clinical trials; thus, this drug perhaps requires additional studies in animal models before its evaluation in patients.

### Flubendazole

Flubendazole is an anthelmintic compound (58); its mechanism of action depends on the disruption of microtubules’ structure and function. This activity attracted considerable interest in the drug as a possible anti-cancer treatment (59); thus, recent studies explored the therapeutic potential of flubendazole against skin cancer (25, 60). For example, a pioneering study conducted by Čáňová et al. (25) demonstrated inhibition of cell growth and proliferation in three distinct types of melanoma cell lines (A375, BOWES, and RPMI-7951), finally leading to caspase-dependent apoptosis. A subsequent report demonstrated that these effects were related to enhanced transcription of p53 and NF-ĸB and phosphorylation of JNK, eventually producing cell cycle arrest and disturbances of the microtubules network (61). Likewise, another study reported that flubendazole suppressed tumor growth and prevented metastasis in mice with xenografts of human melanoma cells (60). According to the authors, those anti-tumor activities were produced by a decrease in STAT3 and PD-1 levels. This drug is not being evaluated in any clinical trial, so its application may need further evaluations in cellular and animal models.

### Itraconazole

Itraconazole is an antimycotic drug commonly utilized worldwide, which has demonstrated the therapeutic potential for skin cancer treatment. In this regard, Kim et al. (62) revealed that itraconazole suppressed the growth of BCC in mice by inhibiting the Hedgehog signaling pathway. This exciting finding provided the foundation for a subsequent Phase II clinical trial in BCC patients (63). The research revealed that the administration of itraconazole *via* oral reduced cell proliferation and tumor area; thus, the authors concluded that itraconazole possesses beneficial effects against BCC in humans. Also, Liang et al. (27) reported that itraconazole inhibited the proliferation of human melanoma cells (A735 and SK-MEL-28 cells) *in vitro*. Interestingly, the drug also suppressed the melanoma growth *in vivo* in a xenograft mice model. Further experiments revealed that the effects were mediated by suppressing Wnt, Hedgehog, and PI3K/mTOR signaling pathways. All these studies provided the basis for clinical trials. In this regard, three clinical trials are studying the effects of itraconazole in patients with skin cancer. Two of them are focused on the molecular effects of locally applied itraconazole on the growth of BCC (NCT02120677 and NCT02735356), whereas the other one is assessing the efficacy and safety of orally administered itraconazole in patients with BBC (NCT02354261).

### Leflunomide

Leflunomide is a compound utilized for the management of rheumatoid arthritis (64). This drug inhibits the enzyme dihydroorotate dehydrogenase (DHODH), which is pivotal in pyrimidine synthesis (65). Since leflunomide impedes the replication of dividing cells, it provided a rationale to propose its use in preclinical cancer studies (66). For example, White et al. (67) explored the possible benefits of utilizing leflunomide to treat skin cancer. They found that leflunomide produced a substantial reduction in melanoma development *in vitro* (RPM17951, A375, and Hs.294T cell lines) e *in vivo* (xenograft in mice). According to the authors, the inhibition of DHODH repressed transcription elongation of genes necessary for melanoma growth such as *myc* and *mitf*. More recent studies have provided more information about molecular targets for leflunomide. For example, O’Donnell et al. (68) stated that the anti-proliferative effects of leflunomide on A375 melanoma cells are dependent on the Aryl Hydrocarbon Receptor. A similar study found that leflunomide caused cell cycle arrest and autophagy through the phosphorylation of Ulk1 and AMP-activated protein kinase (AMPK) in A375 melanoma cells (69). Finally, another study demonstrated that the combination of leflunomide and selumetinib (an inhibitor of MEK) had a synergic effect in reducing BRAFwt and mutant melanoma cells’ proliferation and growth of melanoma tumors in xenografted mice (28). Interestingly, a clinical trial intended to explore the efficacy and safety of the combination of leflunomide and vemurafenib in patients with V600 mutant metastatic melanoma was prematurely terminated due to adverse effects (NCT01611675). Therefore, despite available information about approved drugs, their possible toxicity can be a critical concern in drug repurposing when combined with other substances.

### Mebendazole

Mebendazole is a drug employed to helminths infestation (70), which has also been proposed for drug repurposing in skin cancer (71). A pioneering study exposed that mebendazole produced apoptosis in melanoma cells (30). The apoptotic response was promoted by the phosphorylation of B-cell lymphoma 2 (Bcl-2) and the decrease in X-linked inhibitor of apoptosis (30, 72). Interestingly, the combination of mebendazole, temozolomide, and Bcl-2 antisense had a synergistic effect in inhibiting the growth of two melanoma cell lines (73). Likewise, the combination of mebendazole and trametinib effectively inhibited the proliferation of melanoma cells derived from patients carrying NRAS^mut^/BRAF^WT^ and reduced their growth in xenografted mice (74). Therefore, the concomitant administration of mebendazole with other medications could be an alternative for melanoma treatment. However, this drug has not been assessed in any clinical trial with patients with skin cancer. Thus, its clinical evaluation could require further evidence from preclinical studies

### Metformin

Metformin is a drug commonly used in type 2 diabetes mellitus; it reduces serum glucose levels through diverse mechanisms (75). Notably, melanoma is strongly dependent on glucose metabolism (76), and several epidemiological studies presented a relationship between the use of metformin and lower skin cancer risk (77). Concerning this, an investigation revealed that metformin inhibited tumor growth in mice xenografted with SCC cells (A431 cell line); the effect appeared to be caused by the inhibition of the mTOR and NF-ĸB signaling pathways (78). Similarly, Tomic et al. showed that metformin decreases the proliferation of melanoma cells *in vitro* and reduces tumor growth *in vivo*; those effects were mediated by a cell cycle arrest (31). In comparison, other studies suggested a variety of molecular mechanisms to explain the anti-melanoma properties of metformin, including the decrease of protein TRIB3 expression (79), upregulation of miRNAs expression (80), and induction of immune response in the tumor microenvironment (81). Furthermore, metformin prevented the development of metastasis *in vitro* e *in vivo* by activating the p53 tumor suppressor protein and AMPK (82). Besides, metformin enhanced the anti-proliferative effects of binimetinib (an inhibitor of MEK) in a model of metastatic melanoma cells (83). The molecular mechanism involves P-ERK downregulation and AMPK upregulation. Due to these preclinical pieces of evidence, various clinical studies have been undertaken. Remarkably, at least five clinical trials are ongoing exploring the therapeutic effects of metformin in skin cancer (NCT01638676, NCT01840007, NCT02143050, NCT03311308, and NCT04114136). Although metformin is being studied only as an adjuvant in all the studies.

### Pimozide

Pimozide is an antagonist for dopamine receptors; it is employed to treat Gilles de la Tourette syndrome and schizophrenia (84). Additionally, pimozide has shown promising results for managing several cancers, including skin cancer (36, 85–87). An early clinical trial showed that pimozide might have beneficial effects in patients with formerly medicated metastatic melanoma (86). The possible molecular mechanism for this anti-metastatic effect could be mediated by inhibition of ARPC2, a subunit of the Arp2/3 complex involved in migration and invasion (85). Moreover, preclinical studies demonstrated that the combination of pimozide with other drugs might enhance its anti-melanoma activity. For example, pimozide’s simultaneous use and an inhibitor of indoleamine 2,3-dioxygenase (an enzyme that participates in melanoma tolerance) had a synergistic effect against melanoma in a mouse xenograft model (36). The authors indicated that pimozide inhibited STAT3 and STAT5, regulating tumor immunity. Likewise, Zhao et al. (87) co-administered pimozide and siRNA targeting PD-1 to mice xenografted with melanoma cells. Their results revealed an increase in the anti-tumor effects by inducing apoptosis and enhancing immune function. Lastly, a cutting edge study explored the anti-cancer effects of pimozide and a CpG oligodeoxynucleotide (CpG ODN) on mice xenografted with B16 cells (88). Their results revealed that the combination of those compounds suppressed the melanoma growth and extended experimental subjects’ survival. Those findings were due to the induction of apoptosis, repression of invasion, and enhancement of immune response. Despite all these findings, there are no clinical trials with this drug to date. Those studies shall be necessary to support its repurposing for skin cancer.

### Piroxicam

Piroxicam is a non-steroidal anti-inflammatory compound that blocks the cyclooxygenases-1 and -2 (COX-1 and COX-2) enzymes (89). Several studies have shown that those enzymes participate in the development of actinic keratoses and SCC (90, 91); thus, piroxicam could help their prevention and treatment. In support of this hypothesis, Campione et al. (38) demonstrated that piroxicam’s topical application (1%) had beneficial effects in patients with actinic keratoses. Numerous studies combining piroxicam (0.8%) and sunscreens (SPF 50+) have found very similar results (92–97), which suggests that piroxicam might serve as a chemopreventive agent for SCC. On the other hand, a recent study reported that piroxicam exhibited cytotoxic activity on SCC cells (A431 cell line), highlighting the drug’s therapeutic potential (98). Interestingly, piroxicam had no effects on the proliferation of melanoma A375 cells (99), suggesting that its anti-cancer activity is specific for SCC. Nevertheless, this drug has not been assessed in any clinical trial with patients with SCC; thus, its clinical efficacy has not been proven yet.

## Conclusion and Perspectives

The development of efficacious treatments for skin cancer is costly and time-consuming; hence, old drugs’ repositioning has arisen as an affordable approach. This procedure requires a thorough search through multiple dataset analyses and structure-based virtual screening to select a suitable compound for repurposing (13, 100, 101). Moreover, extensive *in vitro* e *in vivo* analyses are necessary before undertaking clinical trials. In this respect, advances in knowledge of skin cancer cellular and molecular mechanisms have provided essential information for drug repurposing. Likewise, although clinical trial execution usually requires a long time to evidence security and efficacy, the repositioning of drugs for skin cancer will consume less time than the development of novel medications.

Interestingly, even with the evidence for repositioning old drugs for skin cancer, to our knowledge, there is limited evidence from ongoing clinical trials. Possibly, the degree of improvement, and therefore of clinical relevance, does not support the commercial profitability of the discoveries, except for metformin with at least five clinical trial registries, one of them in phase 2. It is noteworthy that metformin, itraconazole, leflunomide, and doxycycline have been proposed as adjuvants, so possibly they would not be one of the primary and first-choice drugs. Nevertheless, the concurrent use of drugs targeting different signaling pathways may enhance their anti-cancer effectiveness, therefore extending the patients’ survival and reducing the relapse risk. Also, this clinical strategy would allow lowering costs related to expensive current anti-cancer medications.

Finally, as in other drug strategies for treating cancers, pharmaceutical technology tools are necessary for adequate administration and effect at skin cancer’s cellular level. In this respect, several nanoformulations can enhance the efficacy of drugs to treat cancer; thus, this approach will allow well-known drugs to be used to treat skin cancer. Although nanosystems for skin cancer are not commercially available, several formulations have been proposed as nanocarriers to effectively deliver known antineoplastic therapeutic agents for skin cancers (102, 103).

## Author Contributions

HC and GL-G conceived the article. HC, OR-H, SA-A, SB-C, GF-G, and GL-G wrote the first draft of the manuscript. MG-T, MG-C, IC-F, and JS-R contributed to the discussion and the search for information. HC, MG-T, MG-C, JS-R, and GL-G critically reviewed the manuscript and edited the final version. IC-F elaborated the **Table 1**. All authors contributed to the article and approved the submitted version.

## Conflict of Interest

The authors declare that the research was conducted in the absence of any commercial or financial relationships that could be construed as a potential conflict of interest.
